# Estimated Therapy Costs and Downstream Cost Consequences of iBASIS–Video Interaction to Promote Positive Parenting Intervention vs Usual Care Among Children Displaying Early Behavioral Signs of Autism in Australia

**DOI:** 10.1001/jamanetworkopen.2023.5847

**Published:** 2023-04-05

**Authors:** Leonie Segal, Jonathan Green, Asterie Twizeyemariya, Kristelle Hudry, Ming Wai Wan, Josephine Barbaro, Teresa Iacono, Kandice J. Varcin, Sarah Pillar, Matthew N. Cooper, Wesley Billingham, Gemma Upson, Andrew J. O. Whitehouse

**Affiliations:** 1 Health Economics and Social Policy Group, Allied Health and Human Performance, University of South Australia, Adelaide, Australia; 2 Division of Neuroscience and Experimental Psychology, University of Manchester, Manchester, United Kingdom; 3 Department of Psychology, Counselling and Therapy, School of Psychology and Public Health, La Trobe University, Melbourne, Victoria, Australia; 4 Division of Psychology and Mental Health, University of Manchester, Manchester, United Kingdom; 5 Olga Tennison Autism Research Centre, School of Psychology and Public Health, La Trobe University, Melbourne, Victoria, Australia; 6 La Trobe Rural Health School, La Trobe University, Bendigo, Victoria, Australia; 7 Menzies Health Institute Queensland, Griffith University, Brisbane, Australia; 8 Telethon Kids Institute, University of Western Australia, Nedlands, Australia

## Abstract

**Importance:**

The growing global prevalence of autism spectrum disorder (ASD) is associated with increasing costs for support services. Ascertaining the effects of a successful preemptive intervention for infants showing early behavioral signs of autism on human services budgets is highly policy relevant.

**Objective:**

To estimate the net cost impact of the iBASIS–Video Interaction to Promote Positive Parenting (iBASIS-VIPP) intervention on the Australian government.

**Design, Setting, and Participants:**

Infants (aged 12 months) showing early behavioral indicators of autism were recruited through community settings into the multicenter Australian iBASIS-VIPP randomized clinical trial (RCT), a 5- to 6-month preemptive parent-mediated intervention, between June 9, 2016, and March 30, 2018, and were followed up for 18 months to age 3 years. This economic evaluation, including cost analysis (intervention and cost consequences) and cost-effectiveness analyses of iBASIS-VIPP compared with usual care (treatment as usual [TAU]), modeled outcomes observed at age 3 through to 12 years (13th birthday) and was conducted from April 1, 2021, to January 30, 2023. Data analysis was conducted from July 1, 2021, to January 29, 2023.

**Exposures:**

iBASIS-VIPP intervention.

**Main Outcomes and Measures:**

To project the diagnostic trajectory and associated disability support costs drawing on the Australian National Disability Insurance Scheme (NDIS), the main outcome was the differential treatment cost of iBASIS-VIPP plus TAU vs TAU and disability-related government costs modeled to age 12 years, using a clinical diagnosis of ASD and developmental delay (with autism traits) at 3 years. Costs were calculated in Australian dollars and converted to US dollars. Economic performance was measured through the following: (1) differential net present value (NPV) cost (iBASIS-VIPP less TAU), (2) investment return (dollars saved for each dollar invested, taking a third-party payer perspective), (3) break-even age when treatment cost was offset by downstream cost savings, and (4) cost-effectiveness in terms of the differential treatment cost per differential ASD diagnosis at age 3 years. Alternate values of key parameters were modeled in 1-way and probabilistic sensitivity analysis, the latter identifying the likelihood of an NPV cost savings.

**Results:**

Of the 103 infants enrolled in the iBASIS-VIPP RCT, 70 (68.0%) were boys. Follow-up data at age 3 years were available for 89 children who received TAU (44 [49.4%]) or iBASIS-VIPP (45 [50.6%]) and were included in this analysis. The estimated mean differential treatment cost was A $5131 (US $3607) per child  for iBASIS-VIPP less TAU. The best estimate of NPV cost savings was A $10 695 (US $7519) per child (discounted at 3% per annum). For each dollar invested in treatment, a savings of A $3.08 (US $3.08) was estimated; the break-even cost occurred at age 5.3 years (approximately 4 years after intervention delivery). The mean differential treatment cost per lower incident case of ASD was A $37 181 (US $26 138). We estimated that there was an 88.9% chance that iBASIS-VIPP would deliver a cost savings for the NDIS, the dominant third-party payer.

**Conclusions and Relevance:**

The results of this study suggest that iBASIS-VIPP represents a likely good-value societal investment for supporting neurodivergent children. The estimated net cost savings were considered conservative, as they covered only third-party payer costs incurred by the NDIS and outcomes were modeled to just age 12 years. These findings further suggest that preemptive interventions may be a feasible, effective, and efficient new clinical pathway for ASD, reducing disability and the costs of support services. Long-term follow-up of children receiving preemptive intervention is needed to confirm the modeled results.

## Key Points

**Question:**

Is iBASIS–Video Interaction to Promote Positive Parenting (iBASIS- VIPP) therapy, a preemptive intervention for infants displaying early behavioral signs of autism, a good societal investment from a third-party payer perspective?

**Findings:**

In this economic evaluation of 89 children with follow-up data from an iBASIS-VIPP randomized clinical trial in Australia, the intervention was estimated to cost A $5131 (US $3607) per child and deliver a cost savings of A $10 695 (US $7519) per child (modeled to age 12 years). In addition, for each dollar invested in treatment, the savings in third-party payer costs was estimated to be A $3.08 (US $3.08).

**Meaning:**

These findings suggest that improvement in child developmental outcomes in the iBASIS-VIPP trial was achieved at an expected net cost savings to the Australian government and that iBASIS-VIPP is a likely good-value societal investment.

## Teaser

This economic evaluation assesses the estimated net costs of the iBASIS–Video Interaction to Promote Positive Parenting intervention vs usual care among children with early behavioral signs of autism in Australia.

## Introduction

Autism spectrum disorder (ASD; autism) is the term for a neurodevelopmental disability characterized by qualitative and lifelong challenges in social interaction and communication as well as the presence of repetitive and sensory behaviors and interests.^1^ Autistic individuals can face barriers to their social and economic participation and well-being.^2^ Impacts vary but can include effects on educational and vocational attainment, mental health, and family functioning.^3,4^

Many countries, including Australia, have reported a marked increase in ASD diagnoses over recent decades, with global prevalence estimates of 1.2% to 2%.^5,6^ Internationally, ASD is a primary cause of years lived with disability (YLD) and is responsible for greater YLD in children than conduct disorder and attention-deficit/hyperactivity disorder combined.^7^ Among male individuals aged 5 to 14 years, ASD ranks in the top 3 causes of YLD in Australia.^8^ As of March 2022, children with an ASD diagnosis made up 54% of all children receiving support through the Australian National Disability Insurance Scheme (NDIS).^9^ The NDIS was established and funded by the Australian government to provide persons with a disability with access to a wide range of services and support related to daily living and to build capacity to encourage independence (eAppendix 1 in Supplement 1). Support can include capital works, technology, and services.

The disability associated with ASD has cost implications for families^10^ and may result in extra government spending on health, education, disability services, and income support. The nature and cost of support services depends on the age of the autistic individual and the level of disability. During childhood, costs are largely associated with the provision of services to support early skill learning, nurture child well-being, and minimize environmental barriers for the child and family. In late adolescence and adulthood, services are commonly required to support employment, independent living, and other community participation activities.^11^ The lifetime support costs for an autistic individual have been estimated at $1.4 million in the US and at £0.92 million (US $1.4 million) in the UK. When an intellectual disability is also present, these costs increase to an estimated $2.4 million and £1.5 million (US $2.2 million), respectively (all at 2011 unit costs).^12^

A challenge for health and disability systems globally is how to apportion finite funding to best support persons with a disability, including autistic individuals, and their families. Optimization of resource allocation requires an understanding of both the efficacy and efficiency (benefits vs costs) of potential interventions, especially to inform when to intervene. The typical clinical pathway for autistic children is to commence the delivery of interventions at the time of diagnosis. Autism emerges in early development, but diagnosis mostly occurs in the late preschool years worldwide.^13^ A new clinical model has recently emerged that commences therapies before diagnosis, when the earliest signs of autism may be present and before full presentation of a diagnosed phenotype. Known as preemptive intervention, this early service response seeks to take advantage of the period of rapid brain development in the first 2 years of life^14^ to improve developmental outcomes across childhood.

The iBASIS–Video Interaction to Promote Positive Parenting (iBASIS-VIPP) intervention is one such preemptive approach that seeks to support infant development by using video feedback techniques to increase caregiver awareness of their infant’s social communication and to guide caregiver responses to build infant social engagement and interaction.^15^ Program evaluation has followed a structured process—an initial determination of acceptability to parents and infants,^15^ followed by a randomized clinical trial (RCT) with 53 infants at increased familial likelihood of ASD.^16^ The RCT results published in 2017 reported a substantial reduction in ASD-related behaviors to follow-up at age 3 years among the cohort who received iBASIS-VIPP in infancy.^16^

A second trial of iBASIS-VIPP^17^ was conducted in Australia, the results of which were published in 2021,^18^ in which the intervention was delivered in the home. This trial tested the efficacy of the intervention in a larger sample of 103 infants with clinical indications showing early behavioral signs of autism assessed via the Social Attention and Communication Surveillance tool.^19^ Recruitment occurred through community settings in Melbourne and Perth, Australia, between June 9, 2016, and March 30, 2018. Nineteen percent of the infants had an older sibling with ASD and 70 (68.0%) were boys. The mean (SD) age was 12.4 (1.9) months for the iBASIS-VIPP intervention group vs 12.38 (2.0) months for the group that received treatment as usual (TAU). Treatment effects replicated those of Green et al,^16^ with a substantial reduction in ASD-related behaviors across the follow-up period to age 3 years among children assigned to iBASIS-VIPP plus TAU (hereinafter the iBASIS-VIPP group) compared with children receiving only TAU,^18^ including notable improvements in parent-rated language outcomes. This trial also reported a lower incidence of children in the iBASIS-VIPP group meeting diagnostic criteria for ASD at 3 years (6.7%) compared with TAU alone (20.5%) (odds ratio, 0.18 [95% CI, 0-0.68]; *P* = .02). To our knowledge, these trials provide the first replicated evidence of a sustained benefit of a preemptive intervention delivered in infancy on ASD-related developmental trajectories.

Policy recommendations rely on a combination of evidence for efficacy and cost-effectiveness—that is, whether observed outcomes represent good value for resources allocated. Consistent with the structured approach for evaluating the iBASIS-VIPP intervention and given evidence of its efficacy and effectiveness (noting a community-based recruitment strategy through maternal and child health nurses and a child development service),^17,18^ we conducted an economic evaluation of the Australian RCT.^17,18^

The aim of this study was to assess whether iBASIS-VIPP represents an efficient use of societal resources, taking a government (insurer) third-party payer perspective and incorporating several performance measures as follows: (1) cost analysis measured by net present value (NPV), (2) cost-effectiveness analysis (cost per outcome of ASD incident cases), (3) timing of break-even cost (child age when intervention cost was matched by downstream cost savings), and (4) dollars saved per dollar invested.

## Methods

### Overview

The iBASIS-VIPP RCT^17,18^ was approved by the human research ethics committees of Princess Margaret Hospital in Perth and La Trobe University in Melbourne, Australia. Each family provided written informed consent.

This economic evaluation was conducted from April 1, 2021, to January 30, 2023, and drew on (1) the Australian RCT^17,18^ to estimate the cost of delivering iBASIS-VIPP and TAU and (2) diagnostic classification at age 3 years (18-month follow-up from program delivery during infancy) to model the diagnostic trajectory to age 12 years (13th birthday).^17,18^ The modeling period was chosen to reflect the quality of evidence on the diagnostic stability of ASD and as a conservative assumption. Downstream support costs were informed by the NDIS.^9^ This study followed the Consolidated Health Economic Evaluation Reporting Standards (CHEERS) statement.

### Differential Intervention Cost

The differential intervention cost (iBASIS-VIPP compared with TAU) incorporated all child development–related services delivered during the 6-month intervention period. Data inputs were derived from trial records.^17,18^ The iBASIS-VIPP intervention cost drew on the record of services delivered by the clinical team, while TAU costs were derived from service use diaries completed by all parents or caregivers during the 5- to 6-month therapy period^17^ and were costed using the Australian Medicare Benefits Schedule in December 2021.^20^

The iBASIS-VIPP intervention is a manualized program with a defined number of sessions delivered by suitably qualified and trained staff in participants’ homes. The intervention cost for delivering iBASIS-VIPP included the following: (1) therapy costs, (2) direct travel costs, (3) therapy-related administration costs, and (4) training and supervision costs. First, therapy costs were calculated as the product of the number of sessions delivered by the therapist (introductory, core, booster) and mean hours per session delivered in participants’ homes, plus other therapy-related activities (eg, time to review video material), administrative time (eg, appointment scheduling), and published clinician hourly rates^21^ (in 2021 Australian dollars) adjusted for client-based hours available, salary on-costs, and overhead. Second, direct travel cost was calculated as the mean distance traveled to deliver sessions in participants’ homes multiplied by the published reimbursement rate per kilometer.^22^ Third, therapy-related administration costs were those undertaken by the therapist, including those mentioned for therapy costs. Finally, training and supervision costs were calculated as the documented number of clinician hours to complete training of a 4-day iBASIS-VIPP workshop, 2 supervised practice cases, plus monthly supervision across the trial, costed at the therapist hourly rate and including trainer or supervisor time. A per-child training cost was calculated by dividing the total training cost by 200 families. This number of families was 4 times that in the iBASIS-VIPP group but was selected to approximate the conditions in a service delivery context, assuming a mean 2-year staff retention.

### Clinical Outcomes

The incidence of ASD diagnostic classification at age 3 years (ASD classification vs none) was a secondary outcome of the trial by Whitehouse et al.^18^ Diagnostic classification was determined by 2 independent clinicians with considerable experience in ASD diagnosis (a clinical psychologist and a speech pathologist) who were blinded to treatment assignment.^18^ The diagnosticians undertook case review, informed by a range of developmental assessments and videos collected at 4 time points from infancy to 3 years, and reached consensus on classification. For this study, a variant of this categorical classification was adopted, allocating children into 3 groups: (1) those who met the *Diagnostic and Statistical Manual of Mental Disorders*, *Fifth Edition* (*DSM-5*),^1^ diagnostic criteria for ASD; (2) children with developmental delay (DD) who had some traits of autism but did not meet *DSM-5* diagnostic criteria for ASD; and (3) all other children. The iBASIS-VIPP intervention was hypothesized to reduce emergent disability, such that fewer children with autism traits would meet the diagnostic threshold for ASD. At the same time, there was no expectation that the combined ASD plus DD group would change. Recognizing that some children described as having DD would meet NDIS eligibility and attract support services, this group was included in the costing model. Percentage-point differences in the incidence of ASD and DD between the iBASIS-VIPP and TAU groups were calculated by simple subtraction, with 95% confidence limits (CLs) reported for ASD but not DD (DD was treated as a residual category, with the program logic suggesting that ASD plus DD would be constant). The best-estimate model assumed that 50% of children classed as having DD would be eligible for NDIS services. To model diagnostic stability to 12 years (13th birthday), we drew on 2 seminal studies reporting the stability of ASD diagnosis from age 3 years into middle childhood.^23,24^ Combining the results of these 2 studies, we adopted an 87% stability of ASD diagnosis to 12 years. We assumed that diagnostic classification would change evenly between ages 3 and 12 years.

The diagnostic trajectory of children not meeting the *DSM-5* criteria for ASD (our DD group) was assumed in the best-estimate model to be unchanged from age 3 years, lacking evidence to inform an alternative assumption. A 10% transition from DD to ASD between ages 3 and 12 years was modeled in the sensitivity analysis.

### Downstream Cost

Expected downstream support costs were estimated based on diagnostic classification (ASD and DD) combined with published NDIS cost data, noting that the NDIS was established to support persons with a disability to have, as far as possible, “the same things in life as other people,”^25^ by providing funds to eligible participants to access a wide range of disability-related support services. The NDIS mean support plan value per child and the percent spent are published quarterly, in data cubes, by disability type and age group (Table 1).^26^ Australian children who have an ASD diagnosis and meet NDIS eligibility typically receive NDIS-funded supports across childhood and into adulthood. In contrast, DD falls within the NDIS Early Intervention Program, whereby access to services beyond age 6 years is essentially dependent on a formal diagnosis consistent with severe disability not supported through mainstream services. Thus, supports for DD were costed until age 6 years. In sensitivity analysis, the transition of some children from DD to ASD was modeled. Mean downstream costs per child in the iBASIS-VIPP and TAU groups, respectively, were calculated by dividing discounted downstream cost estimates for each group by the number of children in each group.

**Table 1.** Mean Annual Support Costs Through the Australian National Disability Insurance Scheme by Primary Diagnosis and Age Group, 2021

Age group, y	Mean payment per child, A$ (US$)^a^	
	Autism spectrum disorder	Developmental delay
0-6	18 360 (12 907)	10 200 (7171)
7-14	15 640 (10 995)	NA
15-18	28 800 (20 247)	NA

Abbreviations: A$, Australian dollars; NA, not applicable.

^a^ Data are mean payments and utilization of plan budgets from the Australian National Disability Insurance Scheme as of March 2022.^26^

### NPV Cost and Return on Investment

To estimate NPV costs (or cost savings), differential treatment costs for iBASIS-VIPP vs TAU across the 6-month intervention period were combined with modeled downstream cost savings to age 12 years and discounted at 3% per annum. We also estimated a return on investment, which comprised dollar savings in downstream costs per dollar invested in iBASIS-VIPP treatment.

### Cost-effectiveness Analysis

We estimated the cost per lower incident case of diagnosed ASD. This was calculated as the differential treatment cost per reduced case meeting the ASD diagnostic criteria at age 3 years.

All costs were calculated in Australian dollars. Costs were then converted to US dollars according to the exchange rate on January 24, 2023 (A $1.00 = US $0.703).^27^

### Sensitivity Analysis

A sensitivity analysis was conducted to explore the estimated NPV cost of plausible adjustments to key parameters (Table 2). The following alternate parameter values were modeled: (1) the proportion of children with an ASD diagnosis at age 3 years at the upper and lower 95% CLs (keeping total ASD plus DD constant), (2) the stability of an ASD diagnosis (95% and 80%, as reported in 2 studies^22,23^), (3) the mean NDIS costs per child (±20% of published cost), (4) the model period (to age 18 years), and (5) the percentage of children in the DD group eligible for NDIS services at 100%. We did not model uncertainty in therapy cost, as economic evaluation is concerned with the association between costs and outcomes. While program costs could change (eg, under an alternate delivery model), the effect on outcomes is unknown. Therefore, the documented costs that delivered the observed outcomes were not adjusted.

**Table 2.** Parameter Values Incorporated Into Probabilistic Sensitivity Analysis to Estimate Net Present Value Cost Savings of iBASIS–Video Interaction to Promote Positive Parenting vs Usual Care

Model parameter	Best estimate	Alternate value
Modeling time frame	Modeled to age <13 y^a^	Modeled to age <18 y^b^
NDIS cost	NDIS mean Australian dollar values for age group and diagnostic category (ASD or DD; Table 1)	±20%
iBASIS-VIPP costs	Mean value based on RCT data (Table 3)	NA^c^
Differential ASD diagnosis incidence at age 3 y meeting NDIS eligibility	Mean value per RCT = −0.138	Normal distribution, based on mean (95% CL, −0.02 to 0.30)
ASD incidence from age 3 y to model end (age 13 or 18 y)	ASD to ASD: 87% diagnostic stability between ages 3 and 12 y (evenly distributed across that age range)	ASD to ASD: age 3-12 y: 80% and 95% stability
		DD to ASD: 10% from age 3 to 12 y (evenly distributed across that age range)^d^
DD incidence at age 3 y meeting NDIS eligibility^e^	50% of children with some ASD features = 0.089^e^	100% of children with DD with some ASD features = 0.178
Differential TAU costs	Mean value from RCT = $118 (Table 3)	NA^c^
Discount	3% per annum	NA

Abbreviations: ASD, autism spectrum disorder; CL, confidence limit; DD, developmental delay; iBASIS-VIPP, iBASIS–Video Interaction to Promote Positive Parenting; NA, not applicable; NDIS, National Disability Insurance Scheme; RCT, randomized clinical trial; TAU, treatment as usual.

^a^ Support costs for eligible children with DD occur up to their seventh birthday as per the Australian NDIS. Beyond that age, a child may be eligible under an alternate disability category, but such uncertainty modeling was not considered appropriate for this study.

^b^ Noting NDIS costs from published annual payments of A $15 640 (US $10 995) per child at ages 7 to 14 years and A $28 800 (US $20 247) per child at ages 15 to 18 years (Table 1).

^c^ Alternate values for iBASIS-VIPP and TAU costs were not modeled because an alternate (lower or higher) cost would mean a different clinical service and as such would potentially impact outcomes and costs and outcomes must be considered together.

^d^ Assumed value (considered plausible based on expert opinion).

^e^ Category of DD was not diagnostic, but rather included children with some features of ASD, not meeting a threshold for an ASD diagnosis. As such, not all of these children would be eligible for (or need) government supports through the NDIS. Therefore, 50% was taken as a reasonable best estimate drawing on expert opinion on knowledge of these children and NDIS eligibility. In sensitivity analysis, it was assumed that 100% of these children would receive supports.

We conducted 1-way sensitivity analyses, reporting the individual effect of modifying each parameter in turn. We also conducted probabilistic sensitivity analysis, describing the combined impact of adjusting all model parameter values simultaneously on NPV cost. In the probabilistic analysis, results are expressed as the percent likelihood that iBASIS-VIPP would be cost saving or achieve any specified cost-savings threshold. Modeling of base-case estimates and 1-way sensitivity analysis was conducted in Excel, version 2302 (Microsoft). Probabilistic sensitivity analysis was performed in TreeAge Pro, version R1.0 (TreeAge Software). Data analysis was conducted from July 1, 2021, to January 29, 2023. The modeling was based on a 2-tailed, 5% *P*-value threshold.

## Results

### Study Participants

Of the 103 infants enrolled in the 2019 iBASIS-VIPP RCT,^17^ 70 (68.0%) were boys and 33 (32.0%) were girls. Follow-up data at age 3 years were available for 89 children who received TAU (44 [49.4%]) or iBASIS-VIPP (45 [50.6%]) and were included in this analysis.

### Cost of Program Delivery

The total cost of iBASIS-VIPP delivery (including TAU services) was estimated at A $5477 (US $3850) per child. For the TAU group, the estimated cost was A $346 (US $243) per child. The cost difference was A $5131 (US $3607) per child. The costs of clinical services, including apportioned training costs, are detailed in Table 3. Further details about training costs are provided in eAppendix 2 in Supplement 1.

**Table 3.** Consultations and Health Care Costs Related to Child Development Services by Group Assignment During the 6-Month Trial Intervention Period, 2021

Intervention	Fee per session, A$ (US$)	Consultation				Differential cost of iBASIS − TAU, A$ (US$)
		TAU group (n = 53)		iBASIS-VIPP + TAU (n = 50)		
		No.	Cost, A$ (US$)	No.	Cost, A$ (US$)	
iBASIS-VIPP						
Clinician time	NA	NA	NA	496	297 972 (209474)^a^	NA
Travel cost reimbursement^b^	NA	NA	NA	NA	7142 (5021)^b^	NA
Consumables	NA	NA	NA	NA	3000 (2109)^c^	NA
Subtotal	NA	NA	NA	496	308 114 (216604)	NA
Mean sessions and cost per child	NA	NA	NA	9.9	4831 (3396)	NA
TAU services (MBS fee^d^)						
Allied health						
Group sessions	38.70 (27.21)	28	1083 (761)	0	NA	NA
Occupational therapy, physiotherapy, or speech therapy or other individual consultation	91.50 (64.32)	163	14 915 (10485)	91	8327 (5854)	NA
Pediatrician	278.75 (195.96)	8	2230 (1568)	2	358 (252)	NA
Psychologist	103.80 (72.97)	1	104 (73)	26	2699 (1897)	NA
Subtotal	NA	200	18 332 (12887)	121	11 384 (8003)	NA
Mean sessions and cost per child	NA	3.8	346 (243)	2.4	228 (160)	NA
Total mean services and cost per child	NA	3.8	346 (243)	12.3	5059 (3556)	NA
Total training costs for iBASIS-VIPP delivery^e^	NA	NA	NA	NA	418 (294)	NA
Total cost per child	NA	NA	346 (243)	NA	5477 (3850)	5131 (3607)

Abbreviations: A$, Australian dollars; iBASIS-VIPP, Video Interaction to Promote Positive Parenting intervention within the British Autism Study of Infant Siblings; MBS, Medicare Benefits Schedule; NA, not applicable; TAU, treatment as usual.

^a^ The mean iBASIS-VIPP consultation cost per session was A $600.75 (US $422.33). This was calculated using a mean 4.5 hours per session, 1.5 hours for program delivery, 1 hour of driving time, and 2 hours of clinical administration time (video review, arranging appointments, and so on) at A $133.50 (US $93.85) per hour. Hourly cost was based on a mean salary of A $92 000 (US $64 676) plus 20% wage on-costs and 30% overhead, 43 working weeks for the occupational therapist for 52 less 4 weeks of annual leave, 2 weeks of public holiday, 2 weeks of sick leave, and 1 week of other leave and assuming 5 hours of clinic-related time per day.

^b^ Mean round-trip distance of 20 km/session and A $0.72 (US $0.51) per kilometer.^22^

^c^ The cost for a laptop computer, video camera plus accessories, and toys was A $750 (US $527) per clinician × 4.

^d^ Accessed in December 2021. A clinician may charge more than the schedule fee; the group workshop session fee is for group therapy by a psychologist. Allied health is MBS item 82010 or item 82020 for an occupational therapist, speech pathologist, or physiotherapist.^20^

^e^ See eAppendix 2 in Supplement 1.

The mean cost for clinical services was A $5059 (US $3556) per child in the iBASIS-VIPP group and A $346 ($243) per child in the TAU group, delivering a mean number of 12.3 and 3.8 services per child, respectively. Costs of training and supervision for iBASIS-VIPP were estimated at A $83 544 (US $58 731) and A $418 (US $295) per child.

### Clinical Outcome

As reported previously by Whitehouse et al,^18^ the incidence of an ASD diagnosis was 20.5% in the TAU group and 6.7% in the iBASIS-VIPP group—or 13.8 percentage points (95% CL, −2% to 30% points) lower for the iBASIS-VIPP group. The observed percentage-point difference was taken as the best estimate for the economic analysis, equivalent to a number needed to treat of 7.2. The proportion of children in the DD category at age 3 years was 37.8% (n = 17) in the iBASIS-VIPP group and 20.5% (n = 9) in the TAU group—17.3% percentage points higher in the iBASIS-VIPP group. Per the program logic, the total percentage of children with autism traits (autism plus DD) was similar in the iBASIS-VIPP and TAU groups at 44.4% (n = 20) and 40.9% (n = 18), respectively.

### Downstream Costs for Disability-Related Services

The best-estimate discounted downstream support costs were A $20 707 (US $14 557) for the iBASIS-VIPP group and A $36 533 (US $25 683) for the TAU group. This was a lower mean cost of A $15 826 (US $11 126) per child for iBASIS-VIPP.

### Economic Performance

#### Cost-effectiveness and NPV

In terms of cost-effectiveness, the estimated cost per reduction in an ASD diagnosis at age 3 years was A $37 181 (US $26 138). When modeled to age 12 years, NPV cost savings were estimated at A $10 695 (US $7519) per child enrolled in iBASIS-VIPP (A $15 826 less A $5131 treatment cost differential; Table 4).

**Table 4.** Downstream Costs and Net Present Value Cost Savings of iBASIS–Video Interaction to Promote Positive Parenting, Best Estimate and 1-Way Sensitivity Analysis

Model parameter	Downstream cost, A$ (US$)	NPV cost savings, A$ (US$)
Best estimate: parameter values as per downstream cost	15 826 (11 126)	10 695 (7519)
NDIS costs + 20% (ASD and DD)	18 991 (13 351)	13 860 (9744)
NDIS costs – 20% (ASD and DD)	12 661 (8901)	7530 (5294)
Differential ASD diagnosis incidence at age 3 y		
High 95% CL	38 562 (27 109)	33 431 (23 502)
Low 95% CL	−5083 (−3573)	−10 214 (−7180)
ASD incidence modeled from age 3 y		
95% diagnostic stability ASD to ASD	16 562 (11 643)	11 431 (8036)
80% diagnostic stability ASD to ASD	15 181 (10 672)	10 051 (7066)
Add 10% DD to ASD at age 12 y, evenly distributed across age range	15 304 (10 759)	10 173 (7152)
At age 3 y, 100% of children described as DD meet NDIS DD criteria = 0.0865 (50% of 0.173)	12 448 (8751)	7317 (5144)
Modeled to age 17 y (18th birthday)	25 110 (17 652)	19 979 (14 045)

Abbreviations: A$, Australian dollars; ASD, autism spectrum disorder; CL, confidence limit; DD, developmental delay; NDIS, National Disability Insurance Scheme; NPV, net present value.

#### Break-Even Cost and Return on Investment

We estimated that the cost of iBASIS-VIPP to the third-party payer (NDIS) would be offset by downstream savings at age 5.3 years, or 4 years after delivery of the preemptive intervention. By age 13 years, we estimated a savings to the third-party payer of A $3.08 (US $3.08) for each A $1.00 (US $1.00) invested in iBASIS-VIPP program delivery (A $15 826 divided by A $5131).

#### Sensitivity Analysis

Results of the 1-way sensitivity analysis are reported in Table 4, and findings of the probabilistic sensitivity analysis are shown in the Figure. The largest impact on estimated NPV cost (savings) was clinical trial outcome, varying from an extra cost of A $10 214 (US $7180 [95% CL]) per child to a cost savings of A $33 431 (US $23 502 [−95% CL]). The NPV estimate was also sensitive to the model period. Modeling to age 17 years (18th birthday) increased the cost savings to A $19 979 (US $14 045). Plausible changes in all other attributes had a smaller impact on estimated NPV cost.

**Figure.**
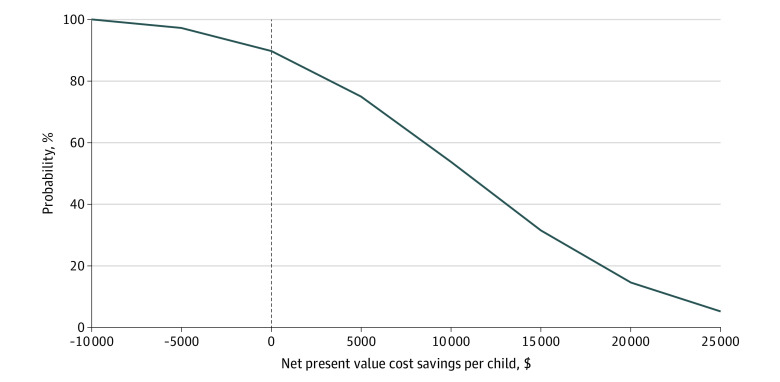
Probabilistic Sensitivity Analysis of Estimated Net Present Value Cost Savings per Child for the iBASIS–Video Interaction to Promote Positive Parenting Intervention vs Usual Care On the x-axis, negative values indicate additional cost and positive values indicate cost savings. Dollars are expressed as Australian dollars. The vertical dashed line indicates the break-even point.

From the probabilistic sensitivity analysis, we estimated an 89% likelihood that NPV is at least 0—that is, downstream cost savings at least equal to intervention cost. This means there was an 88.9% chance that iBASIS-VIPP would deliver costs savings (or no net cost impost) for the NDIS, the dominant third-party payer. The likelihood of generating at least any specified cost savings (read off the x-axis) is described by the y-axis (Figure). For example, we estimated a 74.2% likelihood that NPV cost savings were at least A $5000 (US $3515) (Figure). Further details of the modeling are provided in eAppendices 3 to 11 in Supplement 2.

## Discussion

To our knowledge, this is the first economic evaluation of a preemptive intervention for infants showing early behavioral signs of autism. This study drew on data from a high-quality RCT^17,18^ for evidence on intervention costs and outcomes and on published dedicated government spending through a national disability insurer for downstream cost impacts. The results of this study suggest that iBASIS-VIPP is likely highly cost-effective, with a best-estimate NPV cost savings (intervention cost offset by differential downstream disability support costs) of A $10 695 (US $7519) per enrolled child, an estimated savings of A $3.08 (and US $3.08) for each dollar invested in iBASIS-VIPP. Break-even cost, when disability-related cost savings offset intervention cost, was estimated to occur at age 5.3 years.

A conservative approach to modeling downstream costs was adopted, including only third-party payer costs of the national disability insurer for disability supports and modeled to the 13th birthday. The analysis did not incorporate broader psychosocial and economic impacts (eg, labor force participation among parents^10^) or outcomes related to quality of life for autistic individuals or their family members.^28,29^ If intervention effects were maintained into and through adulthood, the cost savings would be considerably greater—noting the high support costs for autistic adults, which can include disability-based income payments.^12,30^ Under the NDIS, mean annual support payments for adults aged 45 years or older with an ASD diagnosis are more than A $100 000 (US $70 000).^25,26^

There has been considerable discussion within the neurodevelopmental science community about the potential efficacy of preemptive interventions that focus on antecedent neurodevelopmental trajectories,^31,32^ rather than waiting for the emergence of the full behavioral syndrome.^33^ This approach has been advanced through basic science elucidating understandings of the early emergence of autism in the first 2 years of life,^34,35^ and clinical science that has improved developmental surveillance and monitoring of infants for ASD.^19,36,37,38^ These scientific advances, along with knowledge emerging from the neurodiversity movement regarding the importance of adapting the social environment to meet the needs of the autistic child, formed the foundation of the iBASIS-VIPP intervention.^39^ The iBASIS-VIPP intervention is applied within the early developmental epoch and seeks to enrich the social environment and interaction around the infant. The intervention adopts a parent-mediated approach, supporting parents to enhance their skills and apply them in their everyday interactions with their infant, contributing to the relatively modest cost (10 sessions over 5 months) compared with clinician-delivered therapies delivered later in childhood.^40^

There may be potential for improved efficiencies in iBASIS-VIPP delivery, such as in a clinical setting or a combination of clinic, home-based, and telehealth delivery.^41^ However, whether alternate delivery modes would be as effective is unknown. The results of this study suggest that improving our understanding of which infants are most likely to benefit from iBASIS-VIPP would allow the intervention to be targeted with greater specificity, improving economic performance (noting that just 41% of the trial TAU group was described as having autism traits at age 3 years).

An assumption of our model concerned stability of ASD diagnosis—specifically, that 87% of children with an ASD diagnosis at age 3 would carry that diagnosis into middle childhood. This assumption was considered well supported because it was based on 2 high-quality studies.^23,24^ What is less certain is the diagnostic pathway of children in the DD group. Longer-term follow-up of these children is critical to ascertain their trajectories, also noting the higher proportion of children described as having DD in the iBASIS-VIPP group.

The current study has a number of design strengths. The study drew on high-quality clinical research data from an RCT that had 2 years of participant follow-up and replicated findings of a previous RCT. Downstream cost consequences relied on well-characterized published national data from a single-payer disability insurance system, in which the scope of services and supports must relate directly to participants’ disability. As such, payments made under the NDIS provide a reasonably comprehensive estimate of the cost to the human services sector of supporting disability associated with ASD—an approach to the estimation of cost consequences that is not available in other health systems. Estimated benefits are considered conservative in excluding some government, societal, and family costs and impact on quality of life.

### Limitations

This study had a number of limitations. The study population enrolled in the RCT was somewhat advantaged relative to the Australian population in terms of maternal education (60% of parents in the RCT had a bachelor’s degree or higher vs 43% of Australian women aged 25-34 years^42^), English language proficiency, household income, and whether the infant was living with both biological parents (98% vs 89%).^43^ Although these differences did not affect the internal validity of the RCT, they may impact external validity. Noting that study participants were recruited from 2 public health services in Australia and only 11.7% of eligible persons declined enrollment, this may be less of a concern.^19^ In addition, there is mixed evidence concerning whether socioeconomic status moderates the effectiveness of parent-infant therapy, an area for further research.^44,45^ There was a small loss to follow-up from baseline to the final clinical assessment (12.5%), but there were no notable differences in sociodemographic and infant clinical characteristics between the enrolled cohort and those included in the final clinical assessment.

This study estimated potential cost savings in a national setting (Australia), facilitated by the existence of a national disability insurer. The generalizability of these findings to other contexts will depend on service supports available to autistic children. Repeating this study in other jurisdictions would be informative.

## Conclusions

The findings of this economic evaluation, combined with previous clinical trial evidence,^16,18^ suggest that a proactive and developmentally responsive preemptive intervention is efficacious and likely cost-effective in supporting neurodivergent children. Noting the conservative assumptions of our analysis, these findings further suggest that iBASIS-VIPP likely represents a good-value societal investment. Given the considerable potential downstream cost savings, cautious adoption of this preemptive approach is suggested while long-term outcome data are gathered. Although the NDIS is unique to Australia, the support services it provides are similar to those needed by autistic children elsewhere in the world. Given the high and increasing prevalence of ASD globally, identifying preemptive interventions that are efficacious and represent good value is an important input to resource allocation decisions for infants who exhibit early behavioral signs of autism.

## Notes

Supplement 1. **eAppendix 1.** Remit of the Australian National Disability Insurance Scheme (NDIS) **eAppendix 2.** Training and Supervision Costs for 4 Clinicians to Deliver the iBASIS–Video Interaction to Promote Positive Parenting (iBASIS-VIPP) Intervention to 50 Children Across 2 Sites

Supplement 2. **eAppendix 3.** One-way Sensitivity Analysis at Age 12 Years **eAppendix 4.** One-way Sensitivity Analysis at Age 12 Years: Tornado Graphs **eAppendix 5.** Probabilistic Sensitivity Analysis: Input to the Model in TreeAge Pro (Linked to the Model) **eAppendix 6.** Probabilistic Sensitivity Analysis: Decision Tree Image at Age 12 Years **eAppendix 7.** Probabilistic Sensitivity Analysis: Variables Embedded in the Decision Tree **eAppendix 8.** Probabilistic Sensitivity Analysis: Distribution Function Embedded in the Decision Tree **eAppendix 9.** Probabilistic Sensitivity Analysis: Summary Results at Age 12 Years **eAppendix 10.** Probabilistic Sensitivity Analysis: Iteration Results at Age 12 Years **eAppendix 11.** Probabilistic Sensitivity Analysis: Net Cost Savings

Supplement 3.
Data Sharing Statement
