# Errors in Conflict of Interest Disclosures

**DOI:** 10.1001/jamanetworkopen.2024.10357

**Published:** 2024-04-05

In the Original Investigation titled “Estimated Therapy Costs and Downstream Cost Consequences of iBASIS–Video Interaction to Promote Positive Parenting Intervention vs Usual Care Among Children Displaying Early Behavioral Signs of Autism in Australia,”^1^ published April 5, 2023, the conflict of interest disclosure for Dr Whitehouse incorrectly listed him as a joint copyright holder of the iBASIS–Video Interaction to Promote Positive Parenting (iBASIS-VIPP) intervention. Dr Whitehouse is employed by the organization that holds the patent. This article has been corrected.
