# Supplementary material for: Estimated Therapy Costs and Downstream Cost Consequences of iBASIS–Video Interaction to Promote Positive Parenting Intervention vs Usual Care Among Children Displaying Early Behavioral Signs of Autism in Australia
Source: JAMA Netw Open. 2023 Apr 5;6(4):e235847. doi: 10.1001/jamanetworkopen.2023.5847 (PMC10077097; doi:10.1001/jamanetworkopen.2023.5847)
Supplement: Supplement 1. — eAppendix 1. Remit of the Australian National Disability Insurance Scheme (NDIS) eAppendix 2. Training and Supervision Costs for 4 Clinicians to Deliver the iBASIS–Video Interaction to Promote Positive Parenting (iBASIS-VIPP) Intervention to 50 Children Across 2 Sites [file jamanetwopen-e235847-s001.pdf]

# Supplementary Online Content

Segal L, Green J, Twizeyemariya A, et al. Estimated therapy costs and downstream cost consequences of iBASIS–Video Interaction to Promote Positive Parenting intervention vs usual care among children displaying early behavioral signs of autism in Australia. *JAMA Netw Open*. 2023;6(4):e235847. doi:10.1001/jamanetworkopen.2023.5847

**eAppendix 1.** Remit of the Australian National Disability Insurance Scheme (NDIS)

**eAppendix 2.** Training and Supervision Costs for 4 Clinicians to Deliver the iBASIS–Video Interaction to Promote Positive Parenting (iBASIS-VIPP) Intervention to 50 Children Across 2 Sites

This supplementary material has been provided by the authors to give readers additional information about their work.

© 2023 Segal L et al. *JAMA Network Open*.

**eAppendix 1.** Remit of the Australian National Disability Insurance Scheme (NDIS)

"Supports and services delivered for NDIS participants should help people with disability have the same things in life as other people, like somewhere to live, a job, hobbies and the company of families and friends."

"Participants choose and pay for supports and services out of an individually allocated budget based on their goals. Supports and services for participants fall into three categories: core, capital and capacity building.

- Core: A support that helps a participant complete daily living activities.
- Capital: A support for an investment, such as assistive technologies, equipment and home or vehicle modifications, or funding for capital costs (e.g. to pay for Specialist Disability Accommodation).
- Capacity building: A support that helps a participant build their independence and skills."

"Participants receive funding in their plans to access 'reasonable and necessary' services and supports to help pursue their goals. These supports fall into 15 categories aligned with their purpose. These are: 1 Assistance with Daily Life, 2 Transport, 3 Consumables, 4 Assistance with Social & Community Participation, 5 Assistive Technology, 6 Home Modifications, 7 Coordination of Supports, 8 Improved Living Arrangements, 9 Increased Social and Community Participation, 10 Finding and Keeping a Job, 11 Improved Relationships, 12 Improved Health and Wellbeing, 13 Improved Learning, 14 Improved Life Choices, 15 Improved Daily Living."

There are a range of exclusions. "A support will not be funded if it:

- is not related to the participant's disability ....
- relates to day-to-day living costs that are not related to a participant's support needs ...
- can be more appropriately or effectively delivered by another system, such as health or education."

Source: https://www.ndis.gov.au/providers/becoming-ndis-provider/am-i-ready/supports-and-services-funded-ndis

© 2023 Segal L et al. *JAMA Network Open*.

**eAppendix 2.** Training and Supervision Costs for 4 Clinicians to Deliver the iBASIS–Video Interaction to Promote Positive Parenting (iBASIS-VIPP) Intervention to 50 Children Across 2 Sites

| Training item | Descriptive | Hours | Cost[a] AUD |
|---|---|---|---|
| **Clinicians delivering iBASIS-VIPP** | | | |
| Core Training | 4 clinicians x 32 hours[b] | 128 | 13,056 |
| Supervised practice cases to ensure fidelity | 2 practice families per clinician (c) | 360 | 48,060 |
| On-going trial supervision activity | 2 hrs/month x 6 months x 4 clinicians | 48 | 6,408 |
| Sub Total | | *624* | *67 524* |
| **iBASIS-VIPP Supervisor input** | | | |
| Core training delivery | 8 hrs preparation + 30 hrs delivery | 38 | |
| Supervision of practice cases | 2.5 hrs/case x 2 cases x 4 clinicians | 20 | |
| On-going trial supervision activity (to ensure fidelity) | 2 hrs/month/clinician for 6 months | 12 | |
| On-going within-trial certification review of video submissions | 1.5hrs per video[d] | 60 | |
| Subtotal | | ***120*** | ***16,020*** |
| **Total training and supervision cost** | | | ***83,544*** |
| Per child – across 50 children | | | 1,671 |
| **across 200 children[e]** | | | ***418*** |

Notes

a) Hourly cost for clinician/supervisor = AU$133.50 see note (a) to Table 2.
b) 4-day training (30 hour) + 2 hours pre reading.
c) 10 sessions per family @4.5 hrs/session - see note (a) above x 2 families x 4 clinicians.
d) A total of 40 videos were checked for fidelity across the 4 clinicians.
e) To train 4 clinicians and assuming a person who was trained would provide iBASIS-VIPP to 50 children.

© 2023 Segal L et al. *JAMA Network Open*.
