# Supplementary material for: Estimated Therapy Costs and Downstream Cost Consequences of iBASIS–Video Interaction to Promote Positive Parenting Intervention vs Usual Care Among Children Displaying Early Behavioral Signs of Autism in Australia
Source: JAMA Netw Open. 2023 Apr 5;6(4):e235847. doi: 10.1001/jamanetworkopen.2023.5847 (PMC10077097; doi:10.1001/jamanetworkopen.2023.5847)
Supplement: Supplement 3. — Data Sharing Statement [file jamanetwopen-e235847-s003.pdf]

Data Sharing Statement

Segal. Estimated Therapy Costs and Downstream Cost Consequences of iBASIS–Video Interaction to Promote Positive Parenting Intervention vs Usual Care Among Children Displaying Early Behavioral Signs of Autism in Australia. *JAMA Netw Open*. Published April 05, 2023. doi:10.1001/jamanetworkopen.2023.5847

Data
Data available: No

Additional Information
Explanation for why data not available: Trial data is confidential, however, modeling using the data is available as Supplementary Material. Modeling in TreeAge Pro is available by contacting author Dr Twizeyemariya at a.twizeyemariya@gmail.com.
